# The Effect of Age, Stocking Density and Flooring during Transport on Welfare of Young Dairy Calves in Australia

**DOI:** 10.3390/ani4020184

**Published:** 2014-04-11

**Authors:** Ellen C. Jongman, Kym L. Butler

**Affiliations:** 1 Animal Welfare Science Centre, The Melbourne School of Land and Environment, University of Melbourne, Parkville, VIC 3010, Australia; 2 Biometrics Group, Department of Environment and Primary Industries (Victoria), Werribee, VIC 3030, Australia; E-Mail: kym.butler@depi.vic.gov.au

**Keywords:** bobby calves, transport, space allowance, flooring, dairy cattle

## Abstract

**Simple Summary:**

Young calves are vulnerable to the stressful conditions associated with transport. This study examined the effect of age, stocking density and flooring during transport on lying behaviour and some physiological measurements of metabolic state, dehydration and muscle damage in young calves. While physiology and behaviour changed with age, there were no clear effects of transport. More space and straw bedding however was of benefit to calves of all ages, with some indication that straw bedding could ameliorate some of the negative effects of reduced space on lying behaviour.

**Abstract:**

Transport of young (‘bobby’) calves for slaughter is a contentious welfare issue for some sectors of the Australian community. Factors of age, stocking density and flooring need further research to develop appropriate welfare standards for transport of bobby calves. The objective of this study was to identify the space allowance requirements for transport of bobby calves and to understand factors such as age and flooring that minimise risks to calf welfare during transport. Animals aged 3-, 5- and 10-day old were transported for 12 h in a custom-made cattle truck fitted with 9 pens, with movable mesh divisions. Each pen contained 4 calves, with space allowances of 0.2, 0.3 and 0.5 m^2^ per calf and flooring of solid metal, mesh or straw bedding. A total of 432 male dairy calves were transported in 12 trips during the 2-year study. Behavioural measurements included lying during transport, and lying and drinking for 12 h after transport during recovery. Blood samples were taken prior to transport, immediately after transport and 12 h after transport. Blood samples were analysed for metabolic state (glucose, beta-hydroxy-butyrate (BOHB)), hydration (packed cell volume (PCV)) and exhaustion/bruising (creatine kinase (CK) activity). It was found that several measures were affected by age, which indicates that the physiology and in particular lying behaviour of 3-day old calves is fundamentally different from that of older calves. It is unclear how this affects their ability to cope with the stressors of transport. Space affected the posture changes and CK activity during and after transport and it is concluded that space allowance should be at least 0.3 m^2^ per calf for calves of average size, while CK activity suggested that providing more space to 0.5 m^2^ per calf may provide even greater benefits. Straw bedding is of clear benefit to calves during transport, to the extent that it may even reduce some of the negative effects of reduced space on lying behaviour.

## 1. Introduction

In Australia, a bobby calf is defined as a calf not accompanied by its dam and under 4 weeks of age [1]. It is usually a dairy breed or cross, often male, and is destined for slaughter. Most bobby calves are slaughtered within the first week of life.

Because of their age and vulnerability the transport of bobby calves is a contentious welfare issue for some sectors of the Australian community. Overcrowding of bobby calves during transport can result in injury and death, and calves that lie down during transport in overcrowded pens can find it difficult to rise and can become trampled. While there is no law regarding the loading density for bobby calves during transport in Australia, for either short or long duration journeys, the Australian Animal Welfare Standards [1] stipulates that during transport all calves should have sufficient space to lie down on their sternum. It also recommends that all calves less than 30 days old that are transported should have bedding. Calves less than 5 days old travelling without mothers to rearing facilities however must be provided with thick bedding and room to lie down and not be transported for longer than 6 h. These standards are based on the premise that, with bobby calves, extra space and bedding will increase lying and improve welfare. However, there is no experimental evidence to support this premise using behavioural and physiology measures.

Given there is sufficient space, young calves prefer to lie down during transport. In several studies calves were found to lie down 50% [2] to more than 70% [3] of the transport time. Smaller calves may be more affected by transport than bigger calves, as they show a greater need to lie down [4]. 

Mortalities occur during transport and lairage and have been found to increase with transport duration, with truck design being considered a major contributing factor [5]. Although few calves die during transport, Knowles [6] describes an inverse correlation between age at the time of travel and mortality. This effect was particularly noticeable in the weeks after transport, where calves may die due to secondary infections as a consequence of impaired immune function due to transport stress.

Young calves are vulnerable to the stresses of transport and the associated handling during loading and unloading [5,7]. They are particularly hard to handle as a group, as they have not yet developed a herding instinct [8,9]. Even when handled individually, calves become easier to move as their age increases from 3 to 10 days of age [10]. The Welfare Standards for transport for slaughter in Australia [1] states that calves should not be transported for slaughter until they are 5 days old, be fed within 6 h before loading and be transported for no more than 12 h. The guidelines recommend that calves should be of minimum live weight of 23 kg, have hooves that are firm and worn flat and a navel cord that is wrinkled, withered and shriveled. According to the Animal Husbandry Survey 2010, commissioned by Dairy Australia [11], 73% of all farms which sell calves have a permanent record of birth dates and/or a permanent identification tag or collar. However, if calves on farms are not identified by date of birth, it is possible that some calves are transported at an earlier age, as weight and navel cord dryness alone (often used as an indicator of fitness for transport) cannot be used for an indicator of age [12]. 

Factors of age, stocking density and flooring need further research to develop appropriate welfare standards for the transport of bobby calves. The objective of this study was to identify the space allowance requirements for transport of bobby calves and to understand factors such as age and flooring that minimise risks to welfare during transport of bobby calves. The interactions between these factors may lead to better recommendations on the transport of bobby calves of different ages. The effect of transport conditions on the welfare of calves in this study was measured using variables that may indicate effects on metabolic state (glucose, beta-hydroxy-butyrate (BOHB)), hydration (packed cell volume (PCV)) and assessment of muscle exhaustion/bruising (creatine kinase (CK) activity), as have been used in previous studies on transport of calves [2,4,7,13].

## 2. Materials and Methods

This experiment was conducted at the Department of Environment and Primary Industries (DEPI), Werribee Centre, Victoria, Australia with the approval from the DEPI-Victoria Agricultural Research and Extension Animal Ethics Committee and in accordance with the Australian Code of Practice for the Care and Use of Animals for Scientific Purposes [14]. 

### 2.1. Animals

A total of 432 calves (Friesian and Friesian-cross) were transported from commercial farms in Victoria, Australia, where they were kept under commercial conditions in group housing until transport. Each journey consisted of 36 calves sourced from 1 commercial farm in spring or a combination of 4 different farms within close proximity owned by the same farmer in autumn. Transport dates were determined in consultation with each farmer to coincide with dates when large numbers of calves were expected. They were than instructed to keep 36 calves born on certain dates (with 12 calves on each date) in relation to the date of transport. This ensured that a full consignment within three age groups (3, 5 and 10 days old) could be transported on the allocated transport date. Calves were fed 4 L of colostrum in the morning and were loaded onto the truck via a ramp about 1 h after feeding and blood sampling.

### 2.2. Transport

Animals were transported for 12 h in a custom-made cattle truck, where they were protected from the elements as per a standard Australian cattle truck (solid front, ventilation from the sides and no roof). The truck was fitted with 9 pens, with movable mesh divisions so stocking densities could be changed according to the allocated location within the truck. The calves were loaded individually via a portable ramp, and careful handling aimed to reduce stress during loading as much as possible. The route consisted of a mix of country roads, main roads and freeways. A total of 6 trips in spring and 6 trips in autumn were conducted over a 2-year period and no climate extremes of hot, cold or very wet weather were encountered, with temperatures within the range of 10 °C to 25 °C and only an occasional brief shower. All pens could be observed with fixed digital cameras (Ness 2.9 mm transit cameras) and data were recorded on a digital recorder (Ness 8-channel mobile MPEG4 DVR) fitted in the cabin of the truck.

### 2.3. Recovery

After transport, animals were unloaded and housed within one indoor facility in pens in the same groups of 4, with a pen size of 3 m × 3.8 m, where they were observed for another 12 h during recovery. Every pen was observed with fixed digital cameras (b/w CCD infrared cameras fitted with 4.3 mm lenses) and data were recorded on a digital recorder (16-channel I-Watch DVR system) in an adjacent shed. Pen divisions in the shed were made of steel mesh. The animals were provided with straw bedding during recovery from transport. They were not fed during recovery, but did have access to fresh drinking water.

### 2.4. Treatments

The experimental design was a 3 stocking density × 3 age of calf × 3 flooring type factorial, with treatments combinations allocated to trips, and truck position for each trip (see Table 1). The stocking density treatments consisted of space allowances of 0.2 m^2^, 0.3 m^2^ or 0.5 m^2^ per calf. Movable mesh dividers could be placed at different locations to form pens that were 1m wide and either 0.8 m, 1.2 m or 2 m long. Calves were aged 3, 5 and 10 days old at the day of transport. The truck floor consisted of solid steel. A second floor made out of steel mesh (2.5 cm^2^) or a comfortable layer of cereal straw bedding (approximately 10 cm deep) was added on top of the steel floor to the allocated pens so the effect of flooring of either solid steel, steel mesh or straw could be studied. Each pen contained 4 calves, with a total number of 9 pens per trip, with a total of 432 male dairy calves transported over 12 trips during the 2-year study. 

**Table 1:** Diagrammatic representation of the allocation of treatments to trips, and to the truck position for each trip. St denotes straw flooring, So denotes solid flooring and Me denotes mesh flooring. Number of days is the age of calf and the area is the space per calf in a pen of four calves.

	Replicate 1 and 2										
	*Trips 1 and 4*				*Trips 2 and 6*				*Trips 3 and 5*		
	Left	Mid	Right		Left	Mid	Right		Left	Mid	Right
Front	St/3 day/0.2 m^2^	St/5 day/0.3 m^2^	St/10 day/0.5 m^2^		So/10 day/0.3 m^2^	So/3 day/0.5 m^2^	So/5 day/0.2 m^2^		Me/5 day/0.5 m^2^	Me/10 day/0.2 m^2^	Me/3 day/0.3 m^2^
Centre	Me/10 day/0.3 m^2^	Me/3 day/0.5 m^2^	Me/5 day/0.2 m^2^		St/5 day/0.5 m^2^	St/10 day/0.2 m^2^	St/3 day/0.3 m^2^		So/3 day/0.2 m^2^	So/5 day/0.3 m^2^	So/10 day/0.5 m^2^
Rear	So/5 day/0.5 m^2^	So/10 day/0.2 m^2^	So/3 day/0.3 m^2^		Me/3 day/0.2 m^2^	Me/5 day/0.3 m^2^	Me/10 day/0.5 m^2^		St/10 day/0.3 m^2^	St/3 day/0.5 m^2^	St/5 day/0.2 m^2^
	Replicate 3										
	*Trip 7*				*Trip 8*				*Trip 9*		
	Left	Mid	Right		Left	Mid	Right		Left	Mid	Right
Front	St/10 day/0.3 m^2^	St/5 day/0.5 m^2^	St/3 day/0.2 m^2^		St/5 day/0.2 m^2^	St/3 day/0.3 m^2^	St/10 day/0.5 m^2^		St/3 day/0.5 m^2^	St/10 day/0.2 m^2^	St/5 day/0.3 m^2^
Centre	Me/5 day/0.2 m^2^	Me/3 day/0.3 m^2^	Me/10 day/0.5 m^2^		Me/3 day/0.5 m^2^	Me/10 day/0.2 m^2^	Me/5 day/0.3 m^2^		So/5 day/0.2 m^2^	So/3 day/0.3 m^2^	So/10 day/0.5 m^2^
Rear	So/3 day/0.5 m^2^	So/10 day/0.2 m^2^	So/5 day/0.3 m^2^		So/10 day/0.3 m^2^	So/5 day/0.5 m^2^	So/3 day/0.2 m^2^		Me/10 day/0.3 m^2^	Me/5 day/0.5 m^2^	Me/3 day/0.2 m^2^
	Replicate 4										
	*Trip 10*				*Trip 11*				*Trip 12*		
	Left	Mid	Right		Left	Mid	Right		Left	Mid	Right
Front	Me/5 day/0.3 m^2^	Me/10 day/0.2 m^2^	Me/3 day/0.5 m^2^		Me/3 day/0.2 m^2^	Me/5 day/0.5 m^2^	Me/10 day/0.3 m^2^		St/5 day/0.3 m^2^	St/10 day/0.2 m^2^	St/3 day/0.5 m^2^
Centre	St/3 day/0.2 m^2^	St/5 day/0.5 m^2^	St/10 day/0.3 m^2^		St/10 day/0.5 m^2^	St/3 day/0.3 m^2^	St/5 day/0.2 m^2^		So/3 day/0.2 m^2^	So/5 day/0.5 m^2^	So/10 day/0.3 m^2^
Rear	So/10 day/0.5 m^2^	So/3 day/0.3 m^2^	So/5 day/0.2 m^2^		So/5 day/0.3 m^2^	So/10 day/0.2 m^2^	So/3 day/0.5 m^2^		Me/10 day/0.5 m^2^	Me/3 day/0.3 m^2^	Me/5 day/0.2 m^2^

The design had the feature that each calf age and each space allowance were represented once in each left to right position of each trip and represented once in each front to rear position in each trip, so that the main effects of calf age and space could be efficiently estimated. Another feature was that the flooring treatment was represented in all 3 pens of one front to rear position of each trip, so that the flooring main effect was efficiently estimated using between front to rear variation within trips. Other features of the design were that each combination of calf age and space, each combination of calf age and flooring and each combination of space and flooring occurred in one pen in each trip, and each combination of calf age, space and flooring occurred in one pen in each replicate of 3 trips. The randomisation used in creating the design plan for replicates 1, 3 and 4 can be symbolically represented as: Replicate/((Trip/Front to rear position within trips)*(Left to right position across trips)). The symbols ‘/’ and ‘*’ represent nesting and crossing, respectively [15]. 

Apart from a change trip order, the design of replicate 2 is a duplicate of the design of replicate 1.

### 2.5. Measurements

The neck area of the calves was clipped the night prior to transport to expose the jugular vein for ease of blood sampling. At the same time, calves were weighed on portable scales in their home pen. Animals were randomly allocated to transport pens. However, due to concerns for the welfare of the animals, if more than 2 very large calves (45 kg or more) were initially allocated to the smallest pens, then one of those large calves was substituted by a smaller calf. This did affect the kg/pen during the study, with a slightly lower weight in the smaller pens (see Results section). 

Animals were individually identified within each pen by symbols applied with spray marker on their back. In addition, they were fitted with a nylon animal collar around their neck, fastened with Velcro and marked with the same symbols. Each pen was identified with a number, visible on the video recordings.

Calves were observed by video during transport and in the shed for 12 h after transport. Behavioural measurements included lying during transport, and lying and drinking after transport during recovery. Behaviour was observed through continuous sampling, with each transition from standing to lying and vice versa recorded for each calf. While drinking was difficult to observe, when a calf was observed with the head above the drinking bucket it was recorded as ‘drinking’.

Prior to transport and approximately 1 h after feeding in the morning, blood samples were taken via jugular venepuncture, after which the animals were loaded on to the truck and transported for 12 h. Further blood samples were taken immediately after transport and 12 h after transport. Blood samples were analysed for metabolic state (glucose, beta-hydroxy-butyrate (BOHB)), hydration (packed cell volume (PCV)) and assessment of muscle exhaustion/bruising (creatine kinase (CK) activity) by a commercial laboratory (IDEXX Laboratories, Melbourne). Three samples were collected at each blood sampling point: 4-mL serum separator tubes were used to collect blood for analysis for CK activity and BOHB; 2.0-mL fluoride oxalate tubes were used to collect blood for glucose determination; and 4-mL EDTA tubes were used to collect blood for PCV measurement. The whole blood samples were stored at −4 °C before transportation on ice to the laboratory.

### 2.6. Statistical Analysis

With the exceptions of replicates 1 and 2, the unit of analysis was the average of all calves in a pen in a given trip. Corresponding pens (those with the same treatment combination) in trips 1 and 4, trips 2 and 6, and trips 3 and 5 were averaged to form single units of analysis because, other than a randomisation of trip order, the treatments were inadvertently not re-randomised between replicates 1 and 2. Each measurement was analysed using REML mixed model analyses, that in addition to factorial treatment effects, had a fixed effect for replicates and random effects for trip within replicate, left to right position within replicate (but not trip), front to rear position within each trip, and interaction of trip within replicate and front to back within replicate. These particular random effects were included to be in accord with the randomisation used in the experimental design. In accord with standard experimental design principles, when a variance component was estimated to be less than zero, its estimated value was allowed to stand. Some models did not numerically converge, and in these cases some random effect terms were set to be 0 so as to allow numerical convergence. Prior to REML analyses, some measurements were transformed (see Results section) so that the residual variation did not substantially change with the fitted mean and to reduce skewness of the residuals.

For each variable, factorial treatment effects were tested using Wald F tests. Preliminary analyses indicated that there was only weak evidence between age of calves and the space and flooring treatments, and thus P values for the following effects are presented;

(1) Interaction of calf age with space or flooring treatments (*i.e.*, a combined effect of calf age by space, calf age by flooring and calf age by space by flooring interactions) by comparing a model including all treatment combinations with a model including main effects of calf age, space and flooring, and the two-factor interaction of space and flooring,
(2) Effect of calf age by comparing a model including main effects of calf age, space and flooring, and the two-factor interaction of space and flooring with a model including main effects of space and flooring, and the two-factor interaction of space and flooring,
(3) Interaction of space and flooring treatments by comparing a model including main effects of calf age, space and flooring, and the two-factor interaction of space and flooring with a model including main effects of calf age, space and flooring,
(4) Effect of space treatment by comparing a model including main effects of calf age, space and flooring with a model including main effects of calf age and flooring,
(5) Effect of flooring treatment by comparing a model including main effects of calf age, space and flooring with a model including main effects of calf age and space.

In a few cases the denominator degrees of freedom for the Wald F test was not obtainable, and in these cases a Wald chi-squared test was used as a replacement for the Wald F test. Results are presented as predicted means for each calf age and for each combination of space and flooring measurement. The predicted means for calf age are adjusted for combinations of space and flooring treatments and the predicted means for combinations of space flooring treatments are adjusted for the effects of age. Accordingly all the presented predicted means were obtained using a model with fixed effects for replicate, calf age, space, flooring and the interaction of space and flooring, together with the random effects that were used in all models. Back transformed predicted means are calculated for measurements that were transformed prior to REML analysis by performing the inverse of the transformation (e.g., the inverse of square root is squaring, the inverse of log_10_ is the power of 10). Back transformed predicted means are mostly easily interpreted as a predicted median of a pen, but calculated parametrically. All analyses were carried out using the REML mixed models facilities in the GenStat 16 statistical package [15].

## 3. Results

The results indicate that there were effects of calf age on both lying behaviour and several of the metabolic parameters (Table 2). In particular, 3-day-old calves lie down more during transport, as well as during the recovery period. They were also lying down more at the time of arrival of the truck at its destination. Three-day-old calves also showed less interest in drinking water during recovery compared with older calves, although this did not affect their hydration. Physiological measures showed that 3-day-old calves were different from older calves, even before transport. However, there were no interactions between age and space/flooring, so restrictions of space and flooring on lying behaviour affected calves of all ages equally. 

**Table 2:** Effect of age on behaviour and physiology before, during and after transport, and after 12 h of recovery. The interaction *P* value is the *P* value for testing for any interaction between calf age and the space or flooring treatments. Approximate least significant differences can be calculated as the sed multiplied by 2.

Measurement	3 days	5 days	10 days	sed	*P* value	Interaction *P* value
Lying (% of time)	59	48	42	3.1	**0.000**	0.87
√ơLying (% of time)	3.7	3.7	3.8	0.26	0.80	0.40
-Back transformed	13	14	15			
√# ofposture changes	3.7	3.9	3.7	0.17	0.60	0.55
-Back transformed	14.0	14.9	13.6			
Lying at arrival (% of animals)	59	37	36	6.3	**0.000** ^*^	0.095 ^a^
Log_10_(CK)						
-Before transport	2.21	1.97	2.08	0.045	**0.000**	**0.002** ^*^
--Backtransformed (iu/L)	160	90	120			
-Immediately after transport	2.51	2.27	2.37	0.054	**0.001**	0.29 ^b^
--Backtransformed (iu/L)	320	190	230			
-After recovery	2.29	2.11	2.24	0.051	**0.004**	0.86
--Backtransformed (iu/L)	190	130	170			
Glucose						
-Before transport (mmol/L)	6.9	6.1	6.2	0.30	**0.018**	0.49
-Immediately after transport (mmol/L)	6.1	5.8	5.8	0.18	0.058	0.061
-After recovery (mmol/L)	4.5	4.2	4.2	0.16	0.086	0.19
√(BOHB)						
-Before transport	0.38	0.37	0.40	0.014	0.058	0.54
--Backtransformed (mmol/L)	0.15	0.13	0.16			
-Immediately after transport	0.36	0.37	0.40	0.016	**0.032**	0.063
--Backtransformed (mmol/L)	0.13	0.14	0.16			
-After recovery	0.42	0.46	0.49	0.021	**0.006**	0.083
--Backtransformed (mmol/L)	0.18	0.21	0.24			
PCV						
-Before transport (%)	0.42	0.43	0.43	0.009	0.90	**0.013**
-Immediately after transport (%)	0.42	0.41	0.41	0.011	0.59	**0.001**
-After recovery (%)	0.43	0.42	0.42	0.010	0.41	**0.042**
Lying (% of time) during recovery	85	85	81	1.4–1.5	**0.006**	0.59
√# of Lying bouts during recovery	3.1	3.1	3.1	0.09	0.94	0.93
--Backtransformed	9.6	9.5	9.4			
√# of Drinking bouts during recovery	1.0	1.3	1.9	0.15	**0.000**	0.44 ^a^
--Backtransformed	0.9	1.8	3.4			
Weight	37.6	36.6	39.8	0.80	**0.001**	0.39

* Used Wald chi-squared test as calculations for denominator degrees of freedom for the Wald F test did not numerically converge; ^a^ To achieve numerical convergence of models used in calculating test, the Trip within replicate random component was taken to be 0; ^b^ To achieve numerical convergence of models used in calculating test, the Trip within replicate and Front to rear position within trips random components were taken to be 0.

The only measurements with a statistically significant interaction (*P* < 0.05) between calf age and the space and flooring treatments were the logarithm of CK activity before transport and PCV before and immediately after transport and after recovery. The statistically significant result for the logarithm of CK activity before transport must be a chance result because they are sampled prior to transport and prior to animals being randomly allocated to treatment. The PCV results are also likely to be by chance, because the result prior to transport must be by chance, and the subsequent results simply follow the pattern of the first result.

Space did not affect the mean time spent lying, however there was a reduction of the number of posture changes (from lying to standing and vice versa) in truck pens with the lowest space allowance (Table 3). This was also reflected in the significant difference in the standard deviation (sd) in the % of time animals spent lying. In addition, the time spent lying during recovery increased with reduced space allowance during transport. Activity of CK was increased in calves transported at 0.2 m^2^, compared with both 0.3 m^2^ and 0.5 m^2^, with more than double the activity of CK recorded in animals transported at 0.2 m^2^ compared with the other stocking densities. Calves transported at 0.3 m^2^ compared with 0.5 m^2^ also showed some elevated activity of CK. 

**Table 3:** Effect of space allowance and flooring on behaviour and physiology before, during and after transport, and after 12 h of recovery. The standard error of difference between means (sed) differs somewhat depending on which pair of predicted means is being compared, and thus the smallest (min) and largest (max) sed are presented for each measurement. The interaction *P* value is the *P* value for testing for any interaction between flooring and the space treatments. Approximate least significant differences can be calculated as the sed multiplied by 2.

Measurement	Solid			Mesh			Straw			sed		*P* value		
	0.2	0.3	0.5	0.2	0.3	0.5	0.2	0.3	0.5	min	max	Flooring	Space	Interaction
														
Lying (% of time)	43	38	42	54	38	42	58	59	71	5.3	5.9	**0.000**	0.069	0.035
√ơLying (% of time)	4.6	2.9	3.6	5.0	4.2	3.0	3.7	3.5	3.3	0.38	0.42	0.071	**0.001**	**0.011**
-Backtransformed	21	9	13	25	17	9	14	12	11					
√# of posture changes	3.4	4.0	4.0	3.1	4.1	4.4	3.2	4.0	3.8	0.24	0.27	0.43	**0.000**	0.18
-Back transformed	11	16	16	9	17	19	10	16	14					
Lying at arrival (% of animals)	54	24	46	38	29	32	48	64	59	12.1	13.4	**0.016**	0.37	0.14 ^*^
Log_10_(CK)														
-Before transport	2.14	2.02	2.12	2.03	2.03	2.10	2.12	2.00	2.21	0.077	0.079	0.47	**0.030**	0.54 ^*^
--Backtransformed (iu/l)	140	110	130	110	110	120	130	100	160					
-Immediately after transport	2.69	2.33	2.13	2.85	2.40	2.29	2.53	2.16	2.07	0.083	0.091	**0.004**	**0.000**	0.68
--Backtransformed (iu/L)	490	210	140	710	250	190	340	140	120					
-After recovery	2.45	2.19	2.04	2.60	2.26	2.09	2.22	2.04	2.04	0.069	0.080	**0.002**	**0.000**	**0.012**
--Backtransformed (iu/L)	280	150	110	400	180	120	170	110	110					
Glucose														
-Before transport (mmol/L)	6.1	6.8	6.7	6.4	6.0	6.3	6.2	6.4	6.6	0.45	0.50	0.32	0.58	0.63
-Immediately after transport (mmol/L)	5.8	6.1	6.1	5.8	5.6	5.9	6.1	5.8	5.7	0.23	0.28	0.25	0.93	0.33
-After recovery (mmol/L)	4.2	4.9	4.4	4.3	4.1	4.2	4.5	4.2	4.2	0.20	0.25	**0.028**	0.87	0.58
√(BOHB)														
-Before transport	0.40	0.40	0.36	0.38	0.38	0.41	0.40	0.36	0.36	0.026	0.027	0.65	0.67	0.23
--Backtransformed (mmol/L)	0.16	0.16	0.13	0.14	0.14	0.17	0.16	0.13	0.13					
-Immediately after transport	0.43	0.38	0.40	0.35	0.41	0.37	0.34	0.36	0.35	0.024	0.027	**0.009**	0.74	**0.032**
--Backtransformed (mmol/L)	0.19	0.15	0.16	0.12	0.17	0.14	0.11	0.13	0.12					
-After recovery	0.46	0.43	0.44	0.41	0.46	0.48	0.46	0.49	0.47	0.030	0.033	0.31	0.67	0.13
--Backtransformed (mmol/L)	0.21	0.19	0.19	0.17	0.21	0.23	0.21	0.24	0.22					
PCV														
-Before transport (%)	0.43	0.43	0.43	0.43	0.43	0.42	0.42	0.41	0.43	0.015	0.018	0.18	0.77	0.86
-Immediately after transport (%)	0.43	0.42	0.42	0.42	0.42	0.41	0.40	0.40	0.41	0.017	0.020	**0.015**	0.91	0.96
-After recovery(%)	0.43	0.43	0.42	0.43	0.42	0.42	0.41	0.40	0.41	0.016	0.019	**0.022**	0.84	0.97
Lying (% of time) during recovery	87	85	84	85	83	82	84	83	80	1.4	1.5	0.052	**0.049**	0.93
√# of Lying bouts during recovery	3.0	3.3	3.1	2.9	3.0	3.0	3.2	3.1	3.2	0.14	0.16	0.057	0.39	0.56
--Backtransformed	8.7	10.8	9.4	8.6	9.1	9.1	10.1	9.9	10.1					
√# of Drinking bouts during recovery	1.0	1.6	1.5	1.2	1.3	1.2	1.5	1.5	1.5	0.26	0.29	0.41	0.29	0.50
--Backtransformed	1.1	2.7	2.1	1.5	1.8	1.5	2.4	2.3	2.2					
Weight	35.5	38.2	40.6	37.0	38.0	38.6	37.0	38.8	38.3	1.12	1.23	0.93	**0.007**	0.16

* Used Wald chi-squared test as calculations for denominator degrees of freedom for the Wald F test did not numerically converge; ^a^ Left to right position within replicate variance component set to be 0 to allow numerical convergence of model (except for tests of main effects of space and flooring).

Straw bedding had a large effect on lying behaviour, with calves on straw spending more time lying, both as a percentage during transport and at arrival (Table 3). Straw bedding also resulted in lower levels of CK activity and PCV There were some mixed effects of flooring on glucose, with the lowest concentrations of glucose found after recovery in calves transported on mesh floors.

Interestingly, there were several interactions found of space and flooring. In general they indicated that the effect of space was less pronounced when straw bedding was provided, particularly regarding lying behaviour as well as CK activity at 12 h after transport and BOHB immediately after transport.

## 4. Discussion

The results indicate a clear difference in measurements depending on age. However, these effects are apparent even before transport, which indicates that the physiology and particularly lying behaviour of 3-day old calves is fundamentally different from older calves. 

Little research has been conducted on the physiological changes in calves during their first week of life. However, a few studies have included physiological data of young calves, and they show clear effects of age within the first 7 days of life on physiological parameters such as creatin, glucose, NEFA, bilirubin, IGF-I, thyroxine and insulin [16]. In another study similar effects were seen on cortisol, glucose and lymphocyte:neutrophil ratio, which the authors interpreted as immune suppression due to higher levels of cortisol early in life [17]. Indeed, Knowles [6] reported a negative correlation between age at the time of travel and mortality in the weeks after travel in calves less than 4 weeks old, possibly due to impaired immune function as a consequence of transport stress. 

It is unclear if physiological differences across ages affected how well calves coped with the stressors of transport in this study. Since there were no interactions between age and space/flooring, it appears that restrictions of space and flooring affected behaviour and physiology of calves of all ages equally. However, 3-day old calves did lie down more than older calves, which indicates that a comfortable area to lie down is particularly important for these animals. Even though the Australian Animal Welfare Standards and Guidelines for Land Transport of Livestock [1] state that calves should not be transported for slaughter until they are 5 days of age, there is no specific requirement for calves to be identified on farm by their date of birth. While fitness for transport is generally assessed to be represented by a minimum live weight of 23 kg and a dry and shriveled umbilical cord at the junction with the skin, all 3-day-old calves in this experiment had dry, withered navel cords and were over 23 kg live weight. Indeed, anecdotal observations suggest that some calves are transported for slaughter as young as 3 days of age, and greater enforcement of documentation of age on farm may prevent the transportation of calves as young as 3 days of age and may contribute to improved welfare of calves during transport and at slaughter.

Significant effects of space were found, although the percentage of time animals were lying down was not affected. This is in contrast to other studies in which space allowance did affect time spent lying. For example Uetake *et al.* [18] found that increasing the space allowance from 0.25 m^2^ to 0.45 m^2^ increased the number of calves that lie down, although they transported calves that were somewhat older at 11–26 days old, with a body weight of 43–58 kg. Todd *et al.* [13] transported 5–10 day old calves at 0.2 m^2^ and 0.4 m^2^ and also found an increase in lying behaviour with increased space, although those calves were also slightly older and heavier than the current study, which may have inhibited lying at the lower space allowance where space was very limited. Moreover, lying behaviour in the latter study was only casually observed and not specifically measured. Given there is sufficient space, young calves prefer to lie down during transport. In several studies young calves were found to lie down the majority of the time, ranging from 50% [2] to more than 70% [3]. This is similar to the finding in this study, where calves provided with bedding and 0.5 m^2^ laid down 71% of the time. There was a trend in our study for space allowance to affect lying during transport, mainly because this effect was only seen when straw was provided. There was however an increase in lying behaviour during the 12 h recovery after transport in calves that were transported with lower space allowances, indicating that these animals may have been more affected by transport. There was also a clear reduction of the number of posture changes during transport in animals provided with the smallest space allowance. This suggests that once animals were lying down they may have found it more difficult to get up and animals standing may have found it more difficult to lie down. This is also reflected in the higher standard deviation of percentage of time spend lying with the smallest space. In turn, it may explain the higher CK activity found with the smallest space allowance of 0.2 m^2^, although even calves transported at 0.3 m^2^ space allowance had elevated activity of CK compared to calves transported at 0.5 m^2^ space allowances. CK increases in the bloodstream as a result of muscle cell damage resulting from exertion [19] or through direct trauma to the muscle such as bruising [20]. Therefore increased activity of CK may either indicate animals standing becoming muscle fatigued or animals lying down being stood on, resulting in bruising. Bruising is often reported at abattoirs. In a study of 16,400 transported calves, 50% were found to have bruised stifles and it was concluded that this bruising probably occurred during transport [21]. No calves were seen to lose their balance during transport, similarly to Grigor *et al.* [7] who also found that increased space allowance did not result in greater loss of stability or risk of injury. 

Cortisol was not used as a measure of stress in this study. Due to the incomplete development of the HPA axis in young calves the cortisol response is not a reliable measure of stress during transport in these animals [2,22]. 

The Australian Animal Welfare Standards and Guidelines for Land Transport of Livestock [1] stipulate that space allowance during transport should be sufficient for all calves to lie down on their sternum during transport however no specific space allowance is specified. Anecdotally, space allowances as low as 0.17 m^2^ have been observed on commercial transport trucks, even though only a space allowance of 0.3 m^2^ or above provides enough space for all animals to lie down on their sternum. In accordance, the results from this study indicate that space allowance should be at least 0.3 m^2^ per calf for calves of average size, while providing more space to 0.5 m^2^ would provide even greater benefits. Indeed, regulations within the EU requires that transport times of calves less than 14 days old cannot exceed 8 h, are provided with adequate bedding material which guarantees their comfort and space allowances for animals should be at least 0.30 m^2^ to 0.40 m^2^ for a 50 kg calf. These figures may vary, depending not only on the animals' weight and size but also on their physical condition, the meteorological conditions and the likely journey time [23].

While mesh flooring is often provided in trucks specifically set up to transport young calves in order to provide a non-slip and dry surface, no clear advantages were found over a solid floor in this study. However no weather extremes of wet and cold weather, when a mesh floor may have an advantage, were encountered. In fact, higher CK activity found in calves transported on mesh flooring in this study may indicate disadvantages of this flooring. Straw bedding however had a large effect on lying behaviour, with calves on straw spending significantly more time lying. As a result straw bedding resulted in lower activity of CK, indicating that these calves may be less fatigued. The PCV measurements were difficult to interpret as they may have been influenced by factors other than the hydration status of the calves. Acute stressors, by stimulating adrenaline release, can cause splenic contraction thereby increasing the numbers of red blood cells in the circulation [24,25,26]. Glucose concentrations reduced with time as calves were fasted during transport and recovery, as has been reported in previous studies [2,13]. However concentrations stayed within a physiological normal range and did not differ between treatments, other than a slight reduction during recovery in calves transported on a mesh floor. A concurrent increase occurred in BOHB concentration indicating an increase in utilisation of fat reserves for energy, as has also previously been reported [2]. Straw bedding resulted in lower concentrations of BOHB during transport, possibly due to the insulation properties of straw and the lower energy demands while calves were lying down more. The results indicate that calves may be somewhat reluctant to lie down on a metal surface and that this may result in a greater energy expenditure.

Interestingly, there were several interactions of space and flooring found. In general they indicated that the effect of space was less pronounced when straw bedding was provided, overcoming some of the negative effects of reduced space, particularly on lying behaviour. While straw may not be practical under commercial conditions, both from a cost and a biosecurity standpoint, the results indicate that both solid steel or steel mesh are not ideal flooring when transporting bobby calves. Indeed, most European countries require calves up to 2 weeks old to be transported on straw bedding [23]. In addition, in Australia, calves younger than 5 days, who are transported to rearing facilities, must be provided with thick bedding. The guidelines in the Australian Animal Welfare Standards and Guidelines for Land Transport of Livestock [1] also recommend the provision of bedding for calves less than 30 days old to minimise welfare risks. Alternatives such as perforated rubber matting may be investigated to improve the welfare of bobby calves during commercial transport.

## 5. Conclusions

In conclusion, it was found that many measures were affected by age, which indicates that the physiology and particularly lying behaviour of 3-day old calves is fundamentally different from older calves. It is unclear how this affects the calves’ ability to cope with the stressors of transport. However, 3-day old calves did lie down more than older calves, which indicates that a comfortable area to lie down is particularly important for these animals. Space clearly affected the measures during and after transport and the results from this study indicate that space allowance should be at least 0.3 m^2^ per calf for calves of average size, while providing more space to 0.5 m^2^ would provide even greater benefits. Straw bedding is of benefit to calves during transport, to the extent that it may even reduce some of the negative effects of reduced space. While the use of straw may not be practical under commercial conditions, a more suitable surface for calves to lie on during transport should be investigated, such as perforated rubber mats.

## References

[1] (2012). The Australian Animal Welfare Standards and Guidelines for the Land Transport of Livestock. www.animalwelfarestandards.net.au.

[2] Knowles T.G., Warriss S.N., Brown J.E., Edwards J.E., Watkins P.E., Phillips A.J. (1997). Effects of calves less than one month old of feeding or not feeding them during road transport of up to 24 hours. Vet. Rec..

[3] Eicher S.D., Morrow-Tesch J.L. Behavior following subcutaneous electrolyte treatment in transported calves. Proceedings of 34th International Congress of the International Society for Applied Ethology.

[4] Atkinson P.J. (1992). Investigation of the effects of transport and lairage on hydration state and resting behaviour of calves for export. Vet. Rec..

[5] Cave J.P., Callinan A.P.L., Woonton W.K. (2005). Mortalities in bobby calves associated with long distance transport. Aus. Vet. J..

[6] Knowles T.G. (1995). A review of post transport mortality among younger calves. Vet. Rec..

[7] Grigor P.N., Cockram M.S., Steele W.B., Le Sueur C.J., Forsyth R.E., Guthrie J.A., Johnson A.K., Sandilands V., Reid H.W., Sinclair C., Brown H.K. (2001). Effects of space allowance during transport and duration of mid-journey lairage period on the physiological, behavioural and immunological responses of young calves during and after transport. Anim. Sci..

[8] Fraser A.F., Broom D.M. (1997). Farm Animal Behaviour and Welfare.

[9] Thompson K.V. , Kleiman D.G., Allen M.E., Thompson K.V., Lumpkin S. (1996). Behavioral development and play. Wild Mammals in Captivity: Principles and Techniques.

[10] Jongman E.C., Butler K.L. (2013). Ease of moving young calves at different ages. Aus. Vet. J..

[11] Animal Husbandry Survey 2010. http://www.dairyaustralia.com.au/~/media/Documents/Animals%20feed%20and%20environment/Animal%20welfare/Cow%20welfare/DA-Animal-Husbandry-Survey-Dec2010.PDF.

[12] Hides S.J., Hannah M.C. (2005). Drying times of umbilical cords of dairy calves. Aus. Vet. J..

[13] Todd S.E., Mellor D.J., Stefford K.J., Gregor N.G., Bruce R.A., Ward R.N. (2000). Effects of food withdrawal and transport on 5- to 10-day-old calves. Res. Vet. Sci..

[14] (2013). Australian Code for the Care and Use of Animals for Scientific Purposes. http://www.nhmrc.gov.au/guidelines/publications/ea28.

[15] Payne R.W., Weham S., Harding S. (2012). A Guide to REML in Genstat.

[16] Hadorn U., Hammon H., Bruckmaier R.M., Blum J.W. (1997). Delaying colostrum intake by one day has important effects on metabolic traits and on gastrointestinal and metabolic hormones in neonatal calves. J. Nutr..

[17] Jacob S.K., Ramnath V., Philmonia P.T., Raghunandhanan K.V., Kannan A. (2001). Assessment of physiological stress in periparturient cows and neonatal calves. Indian J. Physiol. Pharmacol..

[18] Uetake K., Tanaka T., Sato S. (2011). Effects of haul distance and stocking density on young suckling calves transported in Japan. Anim. Sci. J..

[19] Warriss P.D., Brown S.N., Knowles T.G., Kestin S.C., Edwards J.E., Dolan S.K., Phillips A.J. (1995). Effects on cattle of transport by road for up to 15 hours. Vet. Rec..

[20] Tarrant P.V. (1990). Transportation of cattle by road. Appl. Anim. Behav. Sci..

[21] McCausland I.P., Austin D.F., Dougherty R. (1977). Stifle bruising in bobby calves. N. Z. Vet. J..

[22] Mormede P., Soisson J., Bluthe R., Raoult J., Legarff G., Levieux D., Dantzer R. (1982). Effect of transportation on blood serum composition, disease incidence and production traits in young calves. Ann. Vet. Res..

[23] (2005). European Commission. Regulation (EC) No 1/2005 on the protection of animals during transport. http://eur-lex.europa.eu/LexUriServ/LexUriServ.do?uri=CELEX:32008L0119:EN:NOT.

[24] Potocnik S.J., Wintour E.M. (1996). Development of the spleen as a red blood cell reservoir in lambs. Reprod. Fert. Dev..

[25] Locatelli A., Sartorelli P., Agnes F., Bondiolotti G.P., Picott G.B. (1989). Adrenal response in the calf to repeated simulated transport. Br. Vet. J..

[26] Stewart M., Webster J.R., Stafford K.J., Schaefer A.L., Verkerk G.A. (2010). Technical note: Effects of an epinephrine infusion on eye temperature and heart rate variability in bull calves. J. Dairy Sci..

